# Predictive modeling of miRNA-mediated predisposition to alcohol-related phenotypes in mouse

**DOI:** 10.1186/s12864-018-5004-3

**Published:** 2018-08-29

**Authors:** Pratyaydipta Rudra, Wen J. Shi, Pamela Russell, Brian Vestal, Boris Tabakoff, Paula Hoffman, Katerina Kechris, Laura Saba

**Affiliations:** 1 Department of Biostatistics and Informatics, Colorado School of Public Health, Aurora, 80045 CO USA; 2 Department of Pharmacology, School of Medicine, University of Colorado Anschutz Medical Campus, Aurora, 80045 CO USA; 3 Center for Genes, Environment and Health, National Jewish Health, Denver, 80206 CO USA; 4 Department of Pharmaceutical Sciences, Skaggs School of Pharmacy and Pharmaceutical Sciences, University of Colorado Anschutz Medical Campus, Aurora, 80045 CO USA

**Keywords:** microRNA, Ethanol, Bayesian network, Systems genetics

## Abstract

**Background:**

MicroRNAs (miRNAs) are small non-coding RNAs that bind messenger RNAs and promote their degradation or repress their translation. There is increasing evidence of miRNAs playing an important role in alcohol related disorders. However, the role of miRNAs as mediators of the genetic effect on alcohol phenotypes is not fully understood. We conducted a high-throughput sequencing study to measure miRNA expression levels in alcohol naïve animals in the LXS panel of recombinant inbred (RI) mouse strains. We then combined the sequencing data with genotype data, microarry gene expression data, and data on alcohol-related behavioral phenotypes such as ’Drinking in the dark’, ’Sleep time’, and ’Low dose activation’ from the same RI panel. SNP-miRNA-gene triplets with strong association within the triplet that were also associated with one of the 4 alcohol phenotypes were selected and a Bayesian network analysis was used to aggregate results into a directed network model.

**Results:**

We found several triplets with strong association within the triplet that were also associated with one of the alcohol phenotypes. The Bayesian network analysis found two networks where a miRNA mediates the genetic effect on the alcohol phenotype. The miRNAs were found to influence the expression of protein-coding genes, which in turn influences the quantitative phenotypes. The pathways in which these genes are enriched have been previously associated with alcohol-related traits.

**Conclusion:**

This work enhances association studies by identifying miRNAs that may be mediating the association between genetic markers (SNPs) and the alcohol phenotypes. It suggests a mechanism of how genetic variants are affecting traits of interest through the modification of miRNA expression.

**Electronic supplementary material:**

The online version of this article (10.1186/s12864-018-5004-3) contains supplementary material, which is available to authorized users.

## Background

Non-coding RNAs are defined as biologically functional RNAs that are not translated into proteins. They have a variety of functions including the regulation of the expression of protein coding genes. microRNAs (miRNA) are small non-coding RNAs that bind messenger RNAs (mRNA) and promote degradation or repress translation by post-transcriptional silencing of the target mRNA [1]. The seed region of miRNAs often binds to the 3’ untranslated region (UTR) of the target transcripts to down-regulate their translation [2], but in some cases miRNAs can also positively regulate the mRNA expression [3, 4].

miRNAs have been found to have important roles for many complex traits [5]. The role of miRNAs in alcohol-related phenotypes and addiction is an emerging research area. There is evidence of miRNA expression being associated with both alcohol dependence and alcohol tolerance [6–8] and miRNAs have been suggested to be ‘master regulators’ of alcohol abuse [9]. However, little is known about how miRNAs regulate such complex traits. The influence of genetic background on alcohol-related traits, shown by several behavioral Quantitative Trait Loci (bQTL) studies [10, 11], and on miRNA expression shown by miRNA expression Quantitative Trait Loci (eQTL) studies [12, 13], is well recognized. It is reasonable to propose that some miRNAs may have a mediating role in the genetic predisposition to alcohol-related phenotypes. It can be extremely useful to understand such mediation for developing miRNA-based therapy [14]. The area of miRNA therapeutics is fairly new, but rapidly developing [14–18]. Prediction models that can estimate or infer the trait level based on levels of expression of related miRNAs could help identify therapeutic targets.

There has been some general work on miRNA-mediated effects in the context of a liver gene regulatory network [19], but there is a lack of research on this systems genetics aspect to understand the nature of miRNA-mediated effects in alcohol research. A study of miRNA-mediated genetic effects requires a statistical framework to combine genotype data, miRNA expression data and phenotype data. It is also reasonable to include data on expression of protein-coding genes, since there is increasing evidence of association of both miRNA and gene expression levels with the alcohol-related phenotypes [6, 20–27]. In this work, we propose a method to integrate these different data types to understand the mediating role of miRNAs.

We have used data from a recombinant inbred (RI) mouse panel known as LXS (Inbred Long Sleep × Inbred Short Sleep) [28]. This panel, originally developed to study differential sensitivity to ethanol’s sedative effects, has previously been used for bQTL analysis of various alcohol related traits. We used genotype, miRNA expression, expression of protein coding genes and multiple alcohol-related phenotypes from the LXS mouse panel to understand the nature of the miRNA-mediated genetic effect on predisposition to alcohol-related phenotypes. Because both the miRNA and gene expression level were measured on naïve mice (no ethanol exposure), associations represent predisposing factors rather than responses to alcohol. For the sake of simplicity, we will use the term ‘gene’ to mean ‘protein coding gene’ for the rest of the paper.

The traditional methods [29] to test mediation with potentially multiple mediators (miRNA and gene) have several drawbacks. The most important problem is that it is not possible to learn from the data whether the mediation is serial or parallel, and if assumed serial, what the order of the mediators should be. For example, for a correlated miRNA-gene pair, we do not know for certain if miRNA expression influences gene expression or it is the other way around. The situation can be more complicated when there are multiple miRNAs and/or multiple genes. Bayesian network models can be used to overcome such limitations of a frequentist mediation approach [30]. We have used a Bayesian network analysis to learn the network structure and make predictions. This analysis included necessary adjustments to the usual structure learning methods to make them compatible with our data. The results from such analysis contribute to a better understanding of the miRNA mediated genetic effect.

We report multiple miRNA mediated pathways from genotype to phenotype using Bayesian network analysis. In addition we investigated the genes targeted by the miRNAs and the pathways in which they are enriched. Finally, we have included the predicted changes in the associated phenotypes when the miRNA expression level changes. Such predictions were possible due to the nature of our statistical modeling and can be helpful for future *in silico* testing and targeted drugs.

## Methods

### Animals

The LXS RI panel reported by Markel et al. [31] originally consisted of 77 strains. This sample size is larger than most RI panels, which helps to achieve higher statistical power. The panel was generated from crosses between the ILS (Inbred Long Sleep, abbreviated to L) and ISS (Inbred Short Sleep, abbreviated to S) strains of mice. ILS and ISS mice were reciprocally intercrossed and the F1 mice are intercrossed to produce F2 progeny. Pairs of the F2 mice are then repeatedly inbred to produce the inbred lines (See [32] for details). RI panels have the advantage of being virtually renewable in the sense that they can be genotyped once and used for behavioral, physiological, and molecular phenotyping repeatedly over many generations since mice within a strain can be considered to be genetically identical [33]. This enables us to use existing phenotype data from different sources as long as they correspond to the same LXS strains. However, none of the original datasets from the different sources had all the 77 strains. The genotype data, mRNA expression data, and miRNA expression data consisted of 66, 60, and 59 strains, respectively, with 59 strains belonging to all three. Therefore, when conducting the analysis for each phenotype, we only used the strains that were present in all genomic datasets (i.e. miRNA, mRNA and SNPs) as well as that phenotype data. The list of the strains available for each dataset is reported in Additional file 1: Table S1.

### Alcohol related phenotypes

We have used existing data on three different alcohol-related phenotypes for this study. For the rest of the paper the term ‘phenotype’ denotes ’alcohol-related phenotype’. Descriptive figures showing the distribution of the phenotypes across the strains and the pairwise associations between the three phenotypes are included in the Supplementary Materials (Additional file 1: Figures S1 and S2).

#### Drinking in the dark (DID)

In a free-choice ethanol consumption paradigm, mice are given limited access (for two hours) to 20% ethanol during the early phase of their circadian dark cycle for four consecutive days. The previously published DID phenotype was measured in grams of ethanol per kilogram of body weight in male mice on the third day of alcohol consumption [25]. This phenotype is related to ‘binge drinking’. The DID data consisted of 38 strains, but had 33 strains that were also in the three genomic datasets.

#### Low dose activation (LDA)

For LDA, the mice (male) were given ethanol or saline injections on different days. Difference in total distance traveled in centimeters between the day when ethanol injection (1.8 g/kg) is given and the day when an injection of saline is given was measured as LDA [34]. This phenotype is generally thought to be a measure of sensitivity to low dose of ethanol. The LDA data consisted of 72 strains, but had 57 strains that were also in the three genomic datasets.

#### Loss of righting reflex (LORR)

LORR, also known as Sleep Time, is the phenotype the LS (Long Sleep) and SS (Short Sleep) mice were originally selected for. Mice were given an intraperitoneal dose of ethanol and placed on their backs in a v-shaped tray. LORR was measured by the difference in time (minutes) between the regain of the righting reflex and the time of the initial loss of the righting reflex [35]. Both male and female mice were used for this experiment and the strain means were reported. LORR is a measure of the sensitivity of an animal to the hypnotic effects of a high dose of ethanol. The LORR data consisted of 76 strains, but had 58 strains that were also in the three genomic datasets.

### miRNA expression data

The miRNA expression dataset used was obtained from a subset of the panel with multiple mice per strain. Animal breeding was conducted in the specific pathogen-free facility at the Institute for Behavioral Genetics, Boulder, CO. A total of 175 male mice (59 LXS strains, 2–3 biological replicates per strain) were sacrificed using CO_2_ inhalation followed by decapitation during the light phase and had total RNA extracted from whole brain tissues and fragments between 20–35 bp were selected during the library preparation. Libraries were sequenced on the Illumina HiSeq 2500 platform using single-end 50 base pair reads [36]. The trimmed reads were mapped using a novel k-mer matching method [37] to quantify the number of sequencing reads per individual miRNA. Using this method SNPs for each strain were accounted for in the individual reference miRNAs so that there would not be a mapping bias against those miRNAs. In addition, we used miRDeep2 to identify 362 putative novel miRNAs using the clipped reads and mouse miRBase v20. Following mapping and quantitation, filtering of miRNAs with low counts (less than 5 samples having at least 10 counts), normalization and batch correction were performed [36]. The filtered data consisted of 881 miRNAs including 86 novel miRNAs. A variance stabilizing transformation (VST) [38] was used to transform the read count data to address heteroscedasticity. For our analysis, we collapsed the observations within a strain using the average VST expression across the biological replicates.

### Genotype data

Existing genotype data on the same 59 LXS strains were available from Yang et al. [39]. The genotype data identified approximately 40000 different Single Nucleotide Polymorphisms (SNPs), but only 34642 were informative with different alleles for the parental strains and with no missing data. Many of the 34642 SNPs had the same Strain Distribution Pattern (SDP). Two SNPs are defined to have the same SDP if they are in complete linkage disequilibrium, i.e., there is no recombination in any of the strains between the two SNPs. The data were summarized to 1416 unique SDPs. We used these SDPs for all the statistical analysis but reported the original physical locations of the SNPs corresponding to each SDP. The list of all SDPs and summary of the information on SNPs corresponding to them is reported in Additional file 2: Table S2.

### Gene expression data

For measures of brain mRNA expression levels, the public data set on 60 LXS strains and the two parental strains (*n*=4 to 6 male mice per strain) was downloaded as Affymetrix Mouse Exon 1.0 ST Array (Affymetrix, Santa Clara, CA) CEL files from the PhenoGen website (http://phenogen.ucdenver.edu; [11, 40]). The probe mask described previously in Vanderlinden et al. [11] was used to eliminate low integrity probes, i.e., probes that that did not align uniquely to the mm10 version of the mouse genome or aligned to a region of the genome that harbored a sequence polymorphism between either parental strain and the C57BL/6J reference strain. The remaining probe sets were compared to the Ensembl GRCm38/mm10 version of the transcriptome in mouse. Probe sets targeting the same Ensembl gene were aggregated into a single expression estimate on the log base 2 scale for each sample using the rma-sketch pipeline for normalization and aggregation in Affymetrix Power Tools [41, 42]. Normalized expression estimates were adjusted for batch effects using ComBat [43] and all results are reported at the gene level. For our analysis, we selected the 59 strains for which miRNA data are available and collapsed the RNA expression levels of the individual mice within strains using the average expression level for each strain.

### Statistical analysis

#### Identification of ‘cohesive’ quadruples

**Fig. 1:**
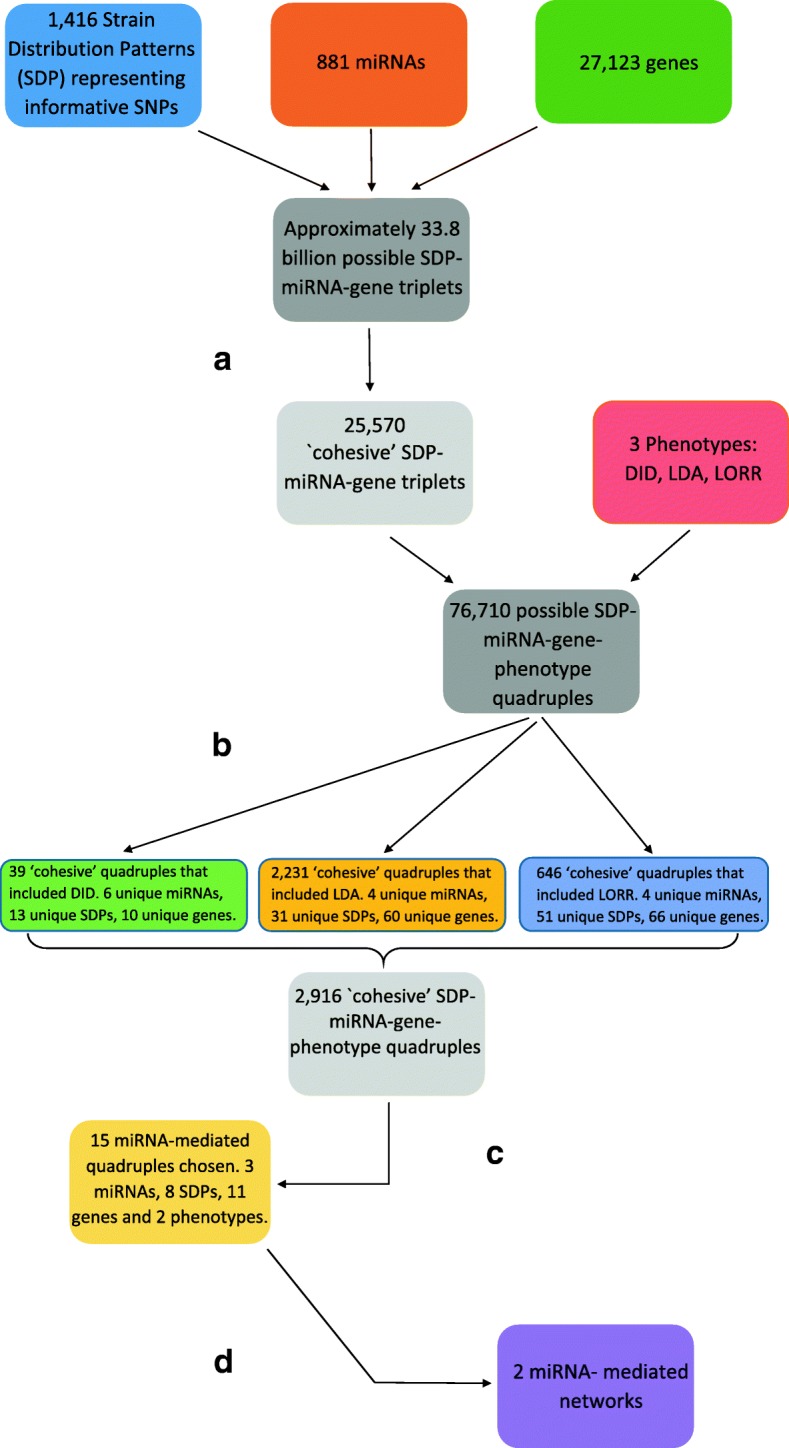
Analytical pipeline to identify miRNA-mediated netowrks associated with alcohol-related phenotypes. The various steps in the flowchart are **a** Select triplets for which all 3 variables are strongly correlated (*p*<10^−3^) with each other; **b** Select quadruples for which the phenotype is significantly associated (*p*<0.05) with each of the 3 components of a chosen ‘cohesive’ triplet; **c** Bayesian Network Analysis separately for each quadruple: Select the quadruple for next step if the best network using the quadruple has a miRNA mediating the effect of the SNP on the phenotype (See details in Fig. 2); **d** Advanced Bayesian networks: miRNAs and genes that were associated with the same phenotype and an SDP from the same region of the genome were combined into larger networks

The summary of the statistical analysis is shown in Fig. 1. We first selected candidate SDP-miRNA-gene triplets for which all the pairwise Pearson correlations between the three variables are strong (nominal *p*-value <10^−3^, step (a) in Fig. 1). We call them ‘cohesive’ triplets. Then we calculated the correlation of the phenotypes with all three variables of the selected candidate triplets. SDP-miRNA-gene-phenotype quadruples for which the phenotype is significantly associated (nominal *p*-value <0.05, step (b) in Fig. 1) with each of the three components of a chosen triplet are selected as ‘cohesive’ quadruples. We used a more stringent *p*-value threshold for the molecular traits since a more direct relationship is expected between them. The list of all ‘cohesive’ quadruples is reported in Additional file 3: Table S3 and the distribution of the correlation between the phenotypes and the ‘cohesive’ triplets is shown by Additional file 1: Figure S3.

**Fig. 2:**
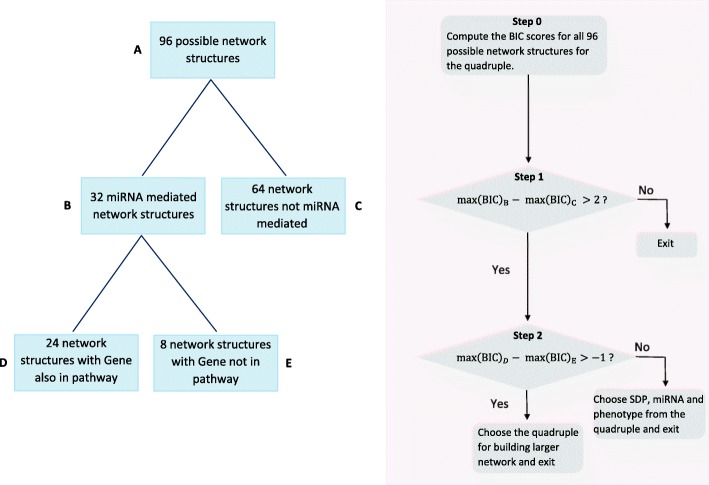
Identification of miRNA mediated quadruples (Step **c** in Fig. 1). For each ‘cohesive’ SDP-miRNA-gene-phenotype quadruple, we perform the following steps. **Step 0:** Compute the BIC scores for all possible network structures that satisfy scientific assumptions. The network structure with the highest score can be considered as the ’most probable’ network for this quadruple. **Step 1:** We choose the SDP, miRNA and Phenotype for building larger network if the quadruple passes the threshold for the BIC-difference in this step. **Step 2:** Also choose the gene for building larger network if the quadruple passes the threshold for the BIC-difference in this step

Due to the large number of correlated variables involved in the analysis, it is extremely difficult to adjust for multiple testing within this filtering step, and it remains an open problem how to make such adjustment. However, we argue that our use of nominal *p*-values in the selection of cohesive quadruples is unlikely to result in many false positives in the final results due to the stringent threshold used in the later stages. We did not use standard genome-wide cutoff since our focus is on identifying miRNA mediating networks, and this is used merely the first step to choose interesting ‘cohesive’ triplets for the subsequent filtering. We evaluated the QQ-plots for all the 3 pairwise associations within a triplet to determine where the points deviated from the diagonal line and chose a significance threshold of 10^−3^, which strikes a balance across all 3 pairwise associations (Additional file 1: Figure S4).

#### Initial Bayesian Network Analysis

Next, Bayesian Network Analysis (BNA) was performed to learn network structure and discover potential miRNA mediated networks (step (c) in Fig. 1). We performed BNA separately for each quadruple. When directing edges within the network we forced any edge with the SDP to be directed away from the SDP and every edge with the phenotype to be directed towards the phenotype. Since SDPs are genetic elements encoded in the mouse genome, it is realistic to use them as ‘causal anchors’ by the principle of Mendelian randomization [44, 45]. The phenotype, on the other hand, can only be a response and cannot affect the miRNA or gene expression since the expression data are obtained from naive mice. There are 96 possible network structures for each quadruple satisfying these properties.

A Bayesian Information Criterion (BIC) score-based network learning procedure was adopted. The BIC score is defined as

1 $$ BIC=\log(Likelihood) - \frac{d}{2}\log(n)  $$

where *n* is the sample size and *d* is the number of parameters of the whole network. A higher value of BIC indicates greater support for the model.

For each quadruple, we performed an exhaustive score-based search across all 96 possible network structures to determine whether the network structure with the highest score has the miRNA as a mediator between the SDP and the phenotype. In order to have a high confidence about the mediating role of the miRNA, we also compared it to the highest score among networks where the miRNA is not a mediator. We chose the SDP, miRNA and phenotype from the current quadruple for the next step only if the BIC clearly favored the model with the miRNA as the mediator, i.e., the difference between the two scores is greater than 2 (Fig. 2, Step 1). It is typically recommended that a difference of 1 in the BIC scores is needed to claim one model to be better than another [46] (Note that BIC as defined in the referenced paper [46] differs from our definition by a constant). If the quadruple met this criterion, we also evaluated the inclusion of the gene within the path from SDP to phenotype. For this comparison, we were more permissive with the difference in BIC scores (Fig. 2, Step 2). The intent of our analysis was to identify miRNA-mediated effects, therefore our criterion for the mediating effect of the miRNA was conservative. With genes, we were more concerned with missing a biologically relevant member of a pathway that has met our initial stringent BIC criteria. For a gene to be included, we compared the highest BIC among networks with both the miRNA and the gene in the path between the SDP and the phenotype to the highest BIC among networks with the miRNA in the path from SDP to phenotype but not the gene. Using a non-inferiority framework, we require the difference in BIC to be greater than −1 for the inclusion of the gene in the final model (Fig. 2, Step 2). These choices of SDP, miRNA, gene and phenotype are not dependent on any optimization algorithm since we conducted exhaustive search among all possible networks.

We used methods for Gaussian Bayesian Network within the **R**-package bnlearn [47] for the purpose of structure learning. It is a common practice to assume that the normalized gene expression and phenotypes follow normal distribution. Also, the miRNA expression obtained from sequencing was transformed using a variance stabilizing transformation [38] which can then be treated as Gaussian. The variable SDP is binary and cannot be transformed to a normal random variable. Therefore, we modified the network learning methods to accommodate hybrid Bayesian networks [48]. The modification of the likelihood is simple due to the fact that the binary random variable SDP is always the causal anchor and the likelihood only involves its unconditional density.

#### Combining chosen quadruples to obtain larger network

Finally, we combined all miRNAs and genes which are associated with the same phenotype and also associated with SDPs from the same region of the chromosome (within 40 megabases from each other) into bigger networks (step (d) in Fig. 1). The SDPs physically located near one another were combined since SNPs that are physically close with each other often show a high Linkage Disequilibrium (LD) pattern and associations of a trait (behavioral or molecular) with multiple such SNPs may just be due the fact that the SNPs are highly correlated with each other. Therefore, we believe that such physically close SDPs are likely to be part of the same larger network. However, the miRNAs and genes did not necessarily have to be physically close to the SDPs. At this final stage the more complex networks are learned using a hill-climbing algorithm [49]. To ensure that the learned structures are stable, we used bootstrapping to repeat the structure learning for 500 bootstraps and used network averaging to combine the results [50, 51]. Network averaging retains an edge and a direction if it appears in more than 50% of the 500 cases. It is possible for some nodes to be not connected to any other nodes in the network, such disconnected nodes are not presented as part of the final network.

#### Finding pathways enriched for genes with binding sites for the mediating miRNAs

Using predicted/validated target databases, we also examined the miRNAs from the final networks to obtain the pathways in which their predicted/validated target genes are enriched. We used the tools multiMiR [52], miRmap [53] and DIANA-miRPath v3.0 [54] for the analysis.

## Results

**Fig. 3:**
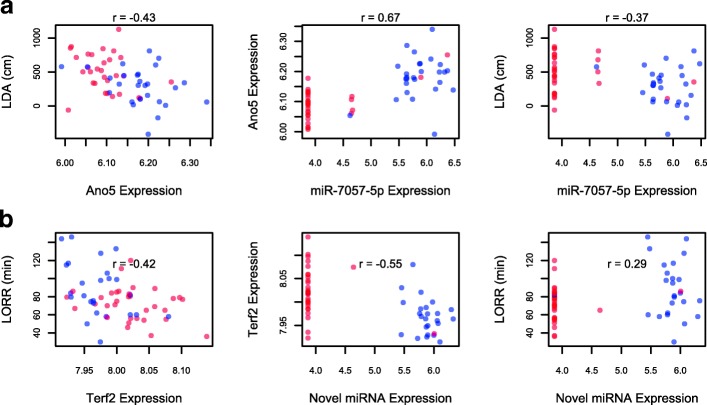
Quantitative relationships between SNP-miRNA-gene-phenotypes quadruples contained within the final network models. Scatter plots of the gene expression (in log base 2 scale) and miRNA expression in the causal pathway with **a** LDA **b** LORR. The color of the points represent the ISS (red) or ILS (blue) alleles for the associated SDP. The value of the correlation coefficient is printed on the top of each scatter plot, the p-values being smaller than the thresholds shown by Fig. 1 in each case

We obtained 2916 candidate ‘cohesive’ quadruples for which the SDP, miRNA, gene and phenotype were strongly associated with each other. Thirty nine quadruples included DID, 2231 included LDA and 646 included LORR. Figure 3 illustrates the association of the four variables (SDP, miRNA, gene and phenotype) with each other for two such quadruples. In the first example, LDA is negatively correlated with the expression of the gene Ano5 (Anoctamin 5) and the miRNA miR-7057-5p, and positively associated with the ISS allele (Fig. 3a). In the second example, LORR is negatively correlated with the expression of the gene Terf2 (Telomeric repeat binding factor 2) and the ISS allele, but positively correlated with the novel miRNA (Fig. 3b).

**Fig. 4:**
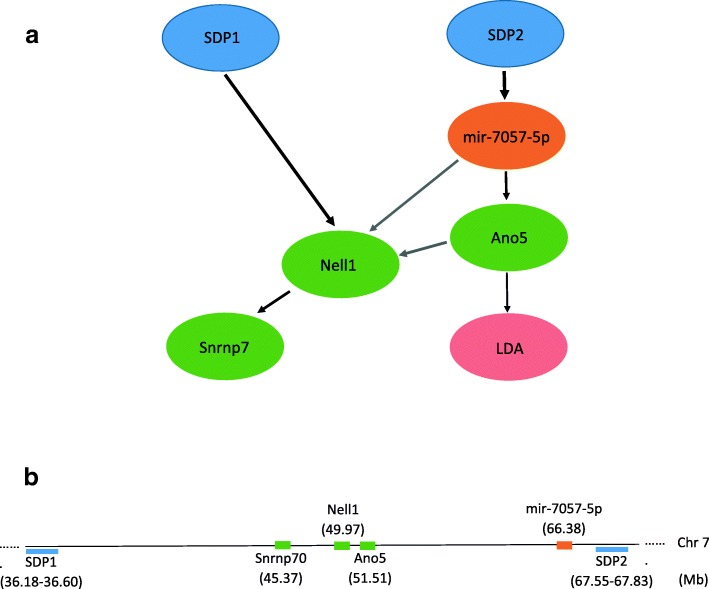
Bayesian network for Low Dose Activation (LDA). **a** A Bayesian network for miRNA mediated genetic effect on LDA. The thickness of the arrows represent the proportion (to scale) of bootstraps for which the edge is present (ranging from 0.53 to 0.99) and the darkness of the arrows represent the proportion of bootstraps for which the edge has the same direction (ranging from 0.54 (gray) to 1 (black)). **b** Illustration of the relative locations of the SDPs, genes and miRNA in the mouse genome (not to scale). The start position of the genes and miRNA are reported. The location of an SDP indicates the range of the original physical locations of the SNPs with the same SDP

Three networks were obtained from our final step of the analysis, of which one was not miRNA-mediated (Additional file 1: Figure S1), when all SDP, miRNA, and genes were included in the model. Of the two miRNA-mediated networks, the first network involves the miRNA miR-7057-5p (Fig. 4), targeting the gene, Ano5, which in turn influences the level of the phenotype LDA. The network also involves the gene Nell1 (Neural EGFL Like 1). The miRNA, the SDP, and the genes are all located on chromosome 7. Predicted target sites for miR-7057-5p in the 3’ UTRs of both Ano5 and Nell1 are found using miRmap [53]. The pathway enrichment analysis found two pathways, Cell Adhesion Molecules and Extracellular Matrix Receptor Interaction, in which the genes predicted to be targeted by the miRNA miR-7057-5p are enriched (Table 1).

**Table 1:** Pathways enriched for genes with known binding sites to miR-7057-5p

miRNA	Pathway	FDR	Genes targeted by miRNA
miR-7057-5p	Cell adhesion molecules	0.0031	Itga6, Itgb2, Cd34, Itgav,
			Cadm3, Itgb1, Pdcd1
	Extracellular Matrix	0.0063	Itga6, Sv2c, Itgav, Itgb1
	Receptor Interaction		

**Fig. 5:**
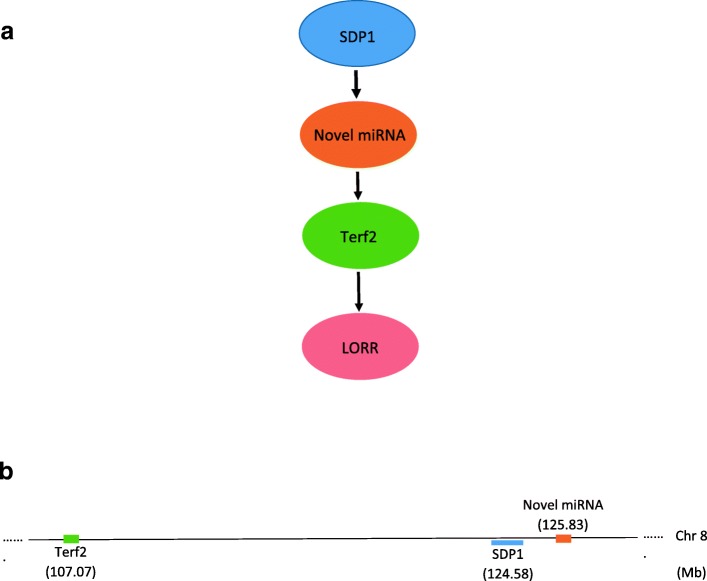
Bayesian network for Loss Of Righting Reflex (LORR). **a** A Bayesian network for miRNA mediated genetic effect on LORR. The thickness of the arrows represent the proportion (to scale) of bootstraps for which the edge is present (ranging from 0.85 to 1) and the darkness of the arrows represent the proportion of bootstraps for which the edge has the same direction (ranging from 0.99 to 1). **b** Illustration of the relative locations of the SDPs, genes and miRNA in the mouse genome (not to scale). The start position of the genes and miRNA are reported. The location of an SDP indicates the range range of the original physical of the SNPs with the same SDP

DIANA-miRPath v3.0 was used for the analysis. No pathways were enriched for the novel miRNA in Fig. 5

The second network (Fig. 5) involves a novel miRNA (mature sequence: CGGGACACCTGAGCTGCCTCTCCT) targeting the gene Terf2, which in turn influences the level of the phenotype LORR. The novel miRNA was not found to be homologous to any other known miRNA upon a homology search using SSEARCH and BLASTN in miRbase (E-value cutoff 1). The miRNA, the SDP, and the gene are all located near one another on chromosome 8 indicating local regulation (i.e. eQTL) of both the miRNA and the mRNA. The proportions of the bootstrap samples where the edges were detected are particularly high (>0.85 for presence and >0.99 for direction of the edges) in the network involving LORR which provides a high confidence about the learned network. The exact values of the proportions are reported in Additional file 1: Figures S6 and S7. For both networks, there are some SDPs that are not connected with any other node in the network, and they are not shown in the final networks.

**Fig. 6:**
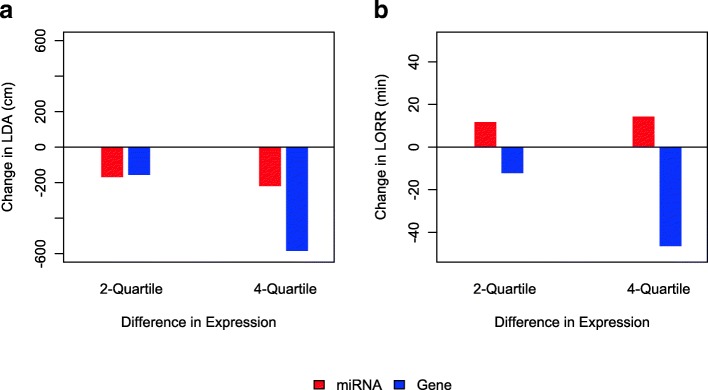
Illustration of prediction based on the fitted Bayesian network models. The figure illustrates the changes in the phenotypes when the expression of the gene or miRNA is increased from the first quartile to the third quartile (2-Quartile difference) or from the minimum to maximum (4-Quartile difference). The bar indicates the change in the phenotype. **a** LDA: The miRNA is miR-7057-5p and the gene is Ano5. **b** LORR. The miRNA is a novel miRNA and the gene is Terf2

Using the fitted Bayesian network models, we determined the predicted changes in the phenotypes for 2-quartile or 4-quartile change in the normalized miRNA or gene expression (Fig. 6). We note that the activation of the corresponding genes reduces the magnitude of both LDA or LORR, i.e. decreases the alcohol sensitivity. However, the corresponding miRNA miR-7057-5p is positively associated with the gene Ano5 for LDA while the novel miRNA is negatively associated with the gene Terf2 for LORR. Therefore, the effect of the mediating miRNA on the phenotype is positive for LORR and negative for LDA.

It is important to note that all of these network structures were learned, and we did not force the phenotype (or any other variable) to have a connected edge in the network. It is possible to not have any miRNA-mediation in the final Bayesian network model even when all the variables are strongly associated. For example, for some of the candidate genes and miRNAs, when the cohesive quadruples were combined for a larger network, the larger network did not have any connected edge with the corresponding phenotype, LORR (Additional file 1: Figure S5).

## Discussion

With the increase in the volume of high-throughput omics data and the advent of less expensive sequencing technologies, more researchers are looking to integrate data of different types to learn more about functional mechanisms. Our statistical framework based on BNA is an effective way to incorporate the different types of data in an unified analysis instead of conducting separate analyses such as miRNA eQTL, gene eQTL and bQTL for alcohol phenotypes. BNA can be considered as a way of decomposing a large joint probability distribution, but it can also serve as a causal probability network model [55]. The method enables us to determine miRNA-mediated predisposition, and the use of BNA ensures that we learn the network structure rather than making prior assumption about the direction of the edges. Although in our final models, the genes, the miRNA, and the SNPs were co-localized, this co-localization was not forced in the procedure and the method should be equally able to detect distal regulations, when present.

Special care was taken to identify the networks for which we have a strong evidence about the mediating role of the miRNA. The thresholds used for BIC differences are arbitrary but commonly used. We used a more stringent threshold for including the mediating miRNA in the final model to make sure that the miRNA is indeed playing a mediating role, while we used a permissive threshold for including a gene to make sure we do not exclude a relevant gene. The use of bootstrap for learning the structure of the larger network minimizes the effect of sampling fluctuation and potential outliers on the final models. We also used a random starting network approach where we used different networks as starting points instead of starting from an empty network [50, 51], and the results were similar (data not shown). The use of bootstrap was not necessary for the final network obtained in Fig. 5 since we could use the exhaustive search and therefore have more confidence about the network learned.

We obtained two different miRNA-mediated networks involving the phenotypes LDA and LORR. No miRNA-mediated networks involving DID was found, which is likely to be due to the much smaller sample size for the DID data (only 33 strains compared to 57 and 58 for LDA and LORR, respectively). Ano5, the gene targeted by the miRNA miR-7057-5p and associated with LDA, encodes a protein which is likely a calcium activated chloride channel (CACC). CACCs are known to be associated with hypnotic ethanol responses in rats after a high dose of ethanol [56]. LDA is also a measure of sensitivity to alcohol, although the effect of high dose and low dose of alcohol could be different. It has been shown by other studies that LORR (sensitivity to high dose) and LDA (sensitivity to low dose) have an inverse relationship, and it is possible that they have similar genetic sources of variation [34]. The predicted target genes for miR-7057-5p are enriched in the pathways ‘Cell Adhesion Molecules’ (CAM) and ‘Extracellular Matrix (ECM) receptor Interaction’. CAMs and ECM Receptors are known to be associated with diseases or nervous system and brain, and addiction including alcohol use disorders [57–60]. The second network involved the gene Terf2 targeted by a novel miRNA and associated with LORR. Terf2 have been shown to have important role in telomere homeostasis and brain development in mice [61]. We have also reported the predicted change in the phenotypes for a fixed change in the normalized miRNA expression or the gene expression (Fig. 6). The direction and magnitude of the predicted change can be helpful for developing targeted drugs.

The results have the same limitations of any in vitro study. However, the results from this paper provide strong candidates for future validation. The stringent threshold for the BIC difference implies high confidence about the mediating role of the miRNAs we reported. For the LORR results, we must assume that the genetic sources of variation in the phenotype are the same in male and female mice since the data contained average measurement for male and female mice while every other dataset were based on male mice only. There is also an amount of uncertainty (of causal SNP) introduced by linkage disequilibrium. The causal SNP may not be uniquely identifiable since many SNPs have the same SDP, and it may require additional studies for more accurate mapping.

## Conclusions

This work enhances association studies by identifying miRNAs that may be mediating the association between SNPs and an alcohol phenotype. We proposed a statistical approach that can identify different mechanisms of how genetic variants are affecting traits of interest through the modification of miRNA expression. In particular, we have identified two miRNA-mediated networks. We also incorporated gene expression data to better understand the functional mechanism and to evaluate alternative drug targets. The ability to detect miRNA-mediated effects and to predict the level of an alcohol related trait based on the miRNA expression provides an opportunity to identify targets for miRNA therapeutics.

## Additional files

Additional file 1 Supplementary Materials. (PDF 1105 KB)

Additional file 2 Supplementary Table 2. (CSV 83 KB)

Additional file 3 Supplementary Table 3. (CSV 470 KB)

## Abbreviations

BIC: Bayesian information criterion
BNA: Bayesian network analysis
bQTL: behavioral quantitative trait Loci
CACC: Calcium activated chloride channel
CAM: Cell adhesion molecules
DID: Drinking in the dark
ECM: Extracellular matrix
eQTL: expression quantitative trait Loci
ILS: Inbred long sleep
ISS: Inbred short sleep
LDA: Low dose activation
LORR: Loss of righting reflex
LS: Long sleep
miRNA: microRNA
mRNA: messenger RNA
RI: Recombinant inbred
SDP: Strain distribution pattern
SS: Short sleep
SNP: Single nucleotide polymorphism
UTR: Untranslated region
VST: Variance stabilizing transformation
