# Supplementary material for: Predictive modeling of miRNA-mediated predisposition to alcohol-related phenotypes in mouse
Source: BMC Genomics. 2018 Aug 29;19:639. doi: 10.1186/s12864-018-5004-3 (PMC6114181; doi:10.1186/s12864-018-5004-3)
Supplement: Supplementary file 1 — Supplementary Materials. (PDF 1105 KB) [file 12864_2018_5004_MOESM1_ESM.pdf]

RESEARCH

Supplementary Materials for Predictive Modeling of miRNA-mediated Predisposition to Alcohol-related Phenotypes in Mouse

Pratyaydipta Rudra[1*], Wen J. Shi[2], Pamela Russell[1], Brian Vestal[3], Boris Tabakoff[4], Paula Hoffman[4,2], Katerina Kechris[1] and Laura Saba[4]

[*]Correspondence:
pratyaydipta.rudra@ucdenver.edu
[1]Department of Biostatistics and Informatics, Colorado School of Public Health, Aurora, CO
Full list of author information is available at the end of the article

Author details
[1]Department of Biostatistics and Informatics, Colorado School of Public Health, Aurora, CO. [2]Department of Pharmacology, School of Medicine, University of Colorado Anschutz Medical Campus, Aurora, CO. [3]Center for Genes, Environment and Health, National Jewish Health, Denver, CO., Denver, CO. [4] Department of Pharmaceutical Sciences, Skaggs School of Pharmacy and Pharmaceutical Sciences, University of Colorado Anschutz Medical Campus, Aurora, CO.

References

Competing interests
The authors declare that they have no competing interests.

Author's contributions
PR constructed the models and performed the statistical analyses. BT, PH, LS and KK designed the LXS miRNA experiments. PHR aligned and quantified the sequencing data and WJS performed the batch effect adjustment. LS and KK supervised the project. All authors discussed the results and provided critical feedback. PR wrote the manuscript with input from all authors.

Funding
Research reported in this publication was supported by the National Institute on Alcohol Abuse (NIAAA) and Alcoholism of the National Institutes of Health (NIH) under award number R01AA021131 and R24AA013162, National Institute on Drug Abuse (NIDA) under award number P30DA044223, and National Library of Medicine Institutional Training Grant, NIH T15LM009451.

Acknowledgements
Not applicable.

Availability of data and materials
Raw data on both miRNA expression and gene expression are available for download at `https://phenogen.ucdenver.edu/`.

Consent for publication
Not applicable.

Ethics approval and consent to participate
Not applicable.

Tables

Genotype	Gene Expression	miRNA Expression	LORR	LDA	DID
LXS100	LXS100	LXS100	LXS100	LXS100	LXS100
LXS101	LXS101	LXS101	LXS101	LXS101	-
LXS102	LXS102	LXS102	LXS102	LXS102	-
LXS103	LXS103	LXS103	LXS103	LXS103	LXS103
LXS107	LXS107	LXS107	LXS107	LXS107	LXS107
LXS110	LXS110	LXS110	LXS110	LXS110	LXS110
LXS112	LXS112	LXS112	LXS112	LXS112	LXS112
LXS114	LXS114	LXS114	LXS114	LXS114	LXS114
LXS115	LXS115	LXS115	LXS115	LXS115	-
LXS122	LXS122	LXS122	LXS122	LXS122	-
LXS123	LXS123	LXS123	LXS123	LXS123	-
LXS13	LXS13	LXS13	LXS13	LXS13	LXS13
LXS14	LXS14	LXS14	-	LXS14	LXS14
LXS16	LXS16	LXS16	LXS16	LXS16	-
LXS19	LXS19	LXS19	LXS19	LXS19	LXS19
LXS22	LXS22	LXS22	LXS22	LXS22	LXS22
LXS23	LXS23	LXS23	LXS23	LXS23	LXS23
LXS24	LXS24	LXS24	LXS24	LXS24	LXS24
LXS25	LXS25	LXS25	LXS25	LXS25	LXS25
LXS26	LXS26	LXS26	LXS26	LXS26	LXS26
LXS3	LXS3	LXS3	LXS3	LXS3	LXS3
LXS32	LXS32	LXS32	LXS32	LXS32	LXS32
LXS34	LXS34	LXS34	LXS34	LXS34	LXS34
LXS36	LXS36	LXS36	LXS36	LXS36	-
LXS39	LXS39	LXS39	LXS39	-	-
LXS41	LXS41	LXS41	LXS41	LXS41	LXS41
LXS42	LXS42	LXS42	LXS42	LXS42	-
LXS43	LXS43	LXS43	LXS43	LXS43	LXS43
LXS46	LXS46	LXS46	LXS46	LXS46	LXS46
LXS48	LXS48	LXS48	LXS48	LXS48	-
LXS49	LXS49	LXS49	LXS49	LXS49	LXS49
LXS5	LXS5	LXS5	LXS5	LXS5	LXS5
LXS50	LXS50	LXS50	LXS50	LXS50	LXS50
LXS52	LXS52	LXS52	LXS52	LXS52	-
LXS60	LXS60	LXS60	LXS60	LXS60	-
LXS64	LXS64	LXS64	LXS64	LXS64	-
LXS66	LXS66	LXS66	LXS66	LXS66	LXS66
LXS7	LXS7	LXS7	LXS7	LXS7	LXS7
LXS70	LXS70	LXS70	LXS70	LXS70	-
LXS72	LXS72	LXS72	LXS72	LXS72	LXS72
LXS73	LXS73	LXS73	LXS73	LXS73	-
LXS75	LXS75	LXS75	LXS75	LXS75	-
LXS76	LXS76	LXS76	LXS76	LXS76	-
LXS78	LXS78	LXS78	LXS78	LXS78	-
LXS8	LXS8	LXS8	LXS8	LXS8	-
LXS80	LXS80	LXS80	LXS80	LXS80	LXS80
LXS84	LXS84	LXS84	LXS84	LXS84	-
LXS86	LXS86	LXS86	LXS86	LXS86	-
LXS87	LXS87	LXS87	LXS87	LXS87	-
LXS89	LXS89	LXS89	LXS89	LXS89	LXS89
LXS9	LXS9	LXS9	LXS9	LXS9	-
LXS90	LXS90	LXS90	LXS90	LXS90	LXS90
LXS92	LXS92	LXS92	LXS92	LXS92	LXS92
LXS93	LXS93	LXS93	LXS93	LXS93	LXS93
LXS94	LXS94	LXS94	LXS94	LXS94	LXS94
LXS96	LXS96	LXS96	LXS96	LXS96	-
LXS97	LXS97	LXS97	LXS97	LXS97	-
LXS98	LXS98	LXS98	LXS98	-	-
LXS99	LXS99	LXS99	LXS99	LXS99	LXS99
LXS28	LXS28	-	LXS28	LXS28	LXS28
LXS51	-	-	LXS51	LXS51	LXS51
LXS68	-	-	LXS68	LXS68	LXS68
LXS10	-	-	LXS10	LXS10	LXS10
LXS56	-	-	LXS56	LXS56	LXS56
-	-	-	LXS2	LXS2	-
-	-	-	LXS31	LXS31	-
-	-	-	LXS38	LXS38	-
-	-	-	LXS54	LXS54	-
-	-	-	LXS55	LXS55	-
-	-	-	LXS59	LXS59	-
LXS62	-	-	LXS62	LXS62	-
-	-	-	LXS79	LXS79	-
-	-	-	LXS88	LXS88	-
-	-	-	LXS117	LXS117	-
LXS35	-	-	LXS35	-	-
-	-	-	LXS124	-	-
-	-	-	LXS61	-	-

Table 1 List of strains available in each dataset. Dash indicates strain not available for the data set.

Figures

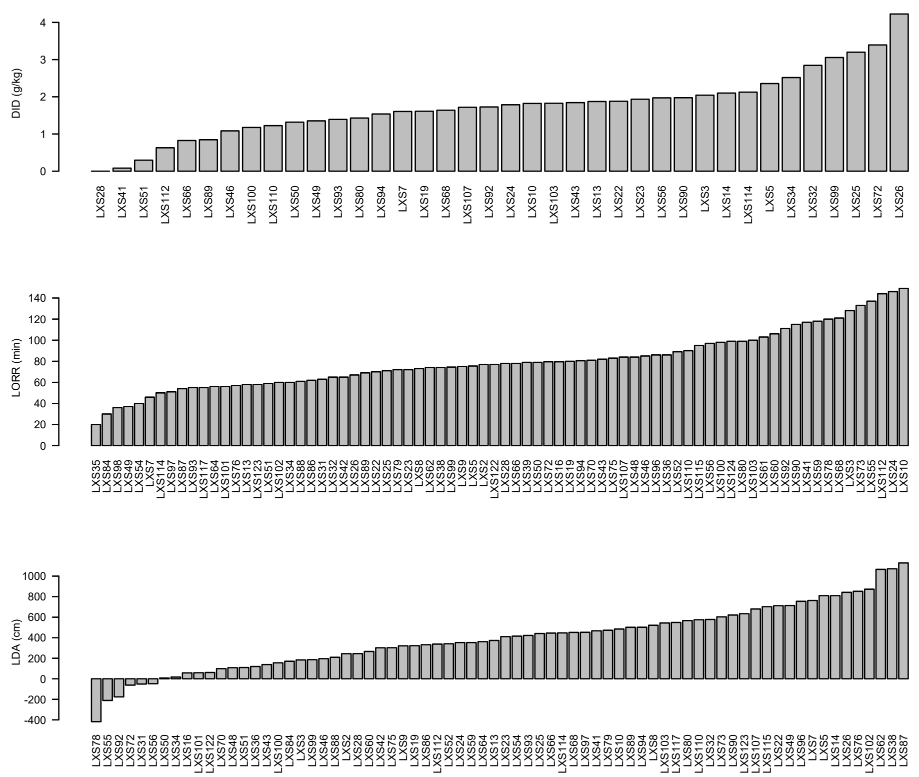

Figure 1 Distribution of the alcohol-related traits. The bar plots illustrate the distribution of the three alcohol-related phenotypes across strains of the LXS recombinant inbred panel. Only the strains that were used for our analysis (present in all the genomic data sets and the corresponding phenotype data set) are shown.

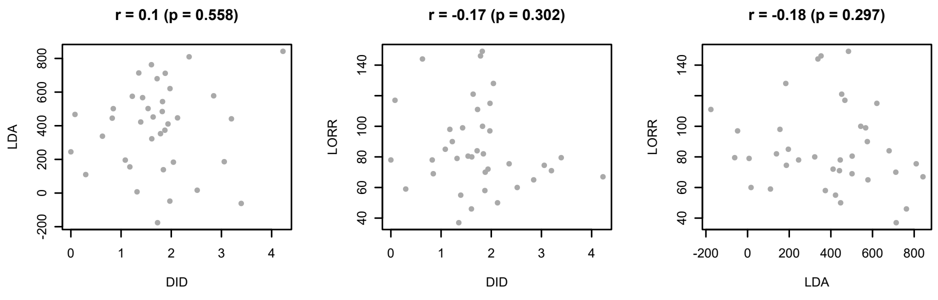

Figure 2 Pairwise association between the alcohol-related traits. Each point represents the within strain mean of each phenotype. The Pearson correlation coefficients and the p-values are reported at the top of each scatter plot.

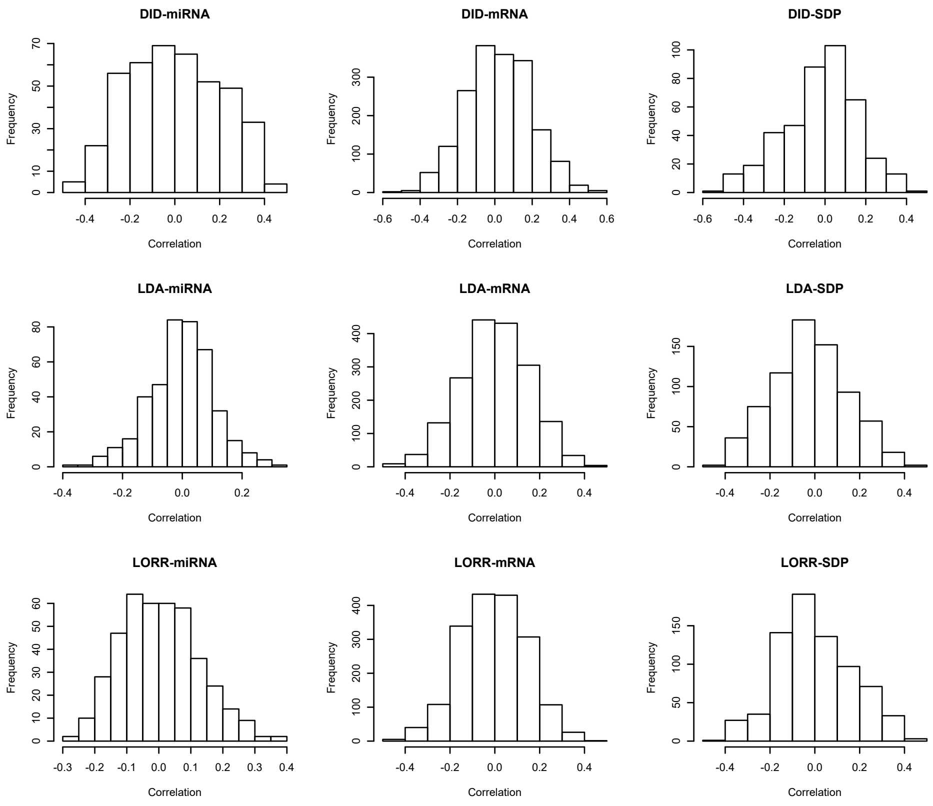

Figure 3 Distribution of the correlation of the alcohol-related traits with the cohesive triplets. The distribution of the Pearson correlation coefficients for pairwise associations between each of the 3 alcohol-related traits and the 3 genomic variables are illustrated by the 9 histograms. Only the 25570 cohesive triplets are included.

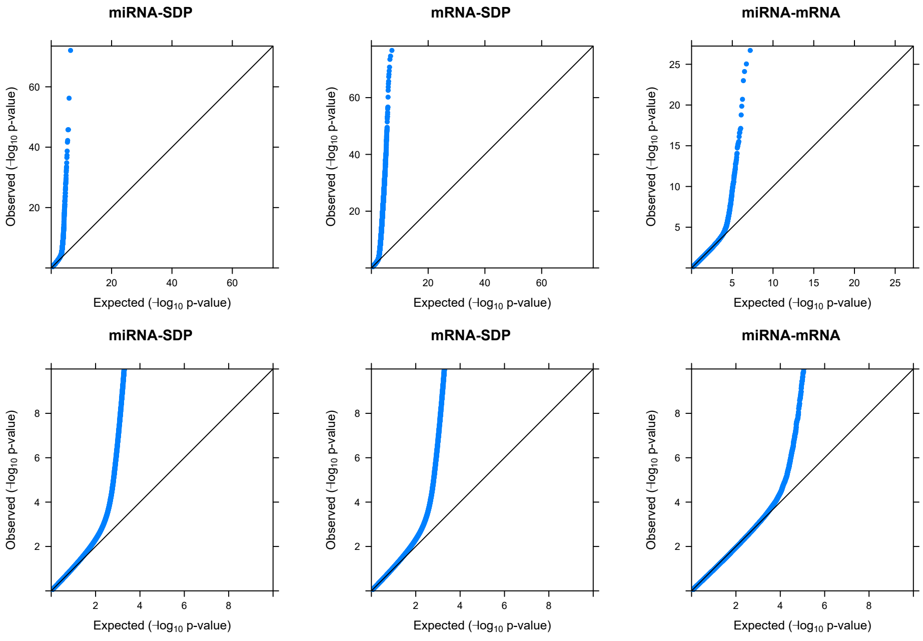

Figure 4 Choice of p-value threshold for identification of cohesive triplets The top row illustrates the observed vs expected p-value QQ-plots ($-\log_{10}$ scale) for all three pairwise associations: miRNA-SDP, mRNA-SDP and miRNA-mRNA. In each case, we only included the SDP/mRNA/miRNAs that have passed the threshold for the other two. In the bottom row, we zoom in on the QQ-plots so that the more extreme p-values are omitted for a better view of where points are deviating from the diagonal line. We chose the p-value threshold 10^{-3} which is an intermediate value for the point of deviation for all three comparisons.

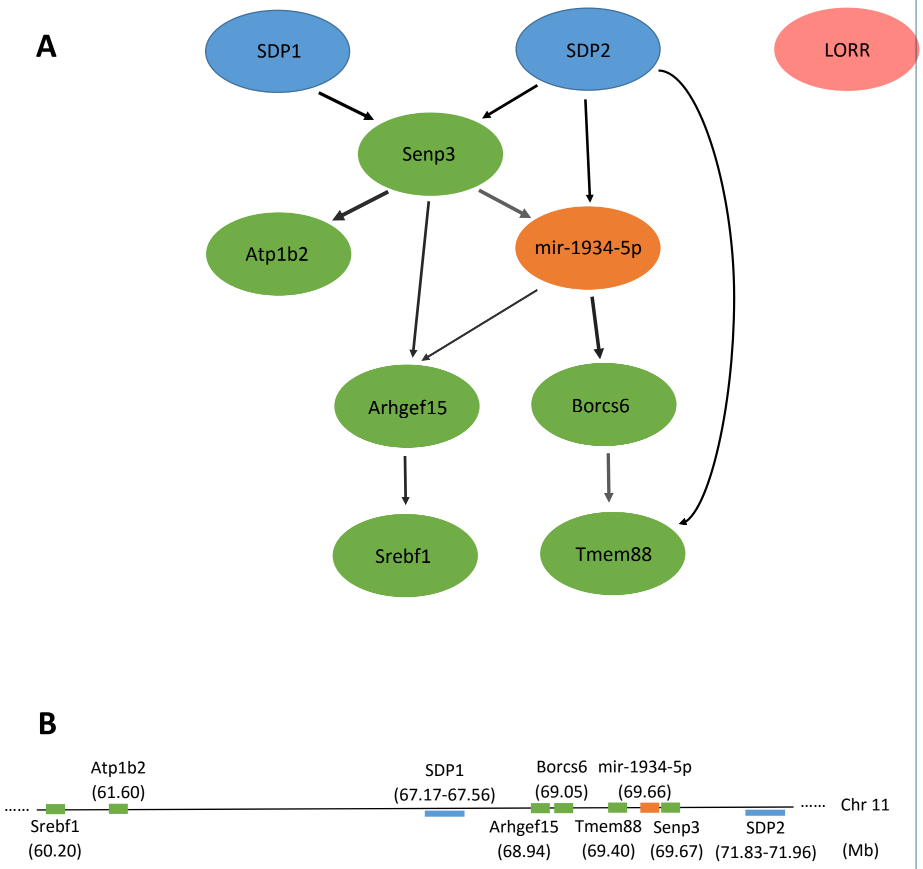

Figure 5 Bayesian network for LORR. A. A Bayesian network using the candidate SDP, miRNA and genes related to LORR. However, LORR was not part of the final network. The thickness of the arrows represent the proportion of bootstraps for which the edge is present (ranging from 0.54 to 1) and the darkness of the arrows represent the proportion of bootstraps for which the edge has the same direction (ranging from 0.63 to 1). **B**. Illustration of the relative locations of the SDPs, genes and miRNA in the mouse genome (not exact scale). The start position of the genes and miRNA are reported. The location of an SDP indicate the range of locations of the SNPs having that SDP.

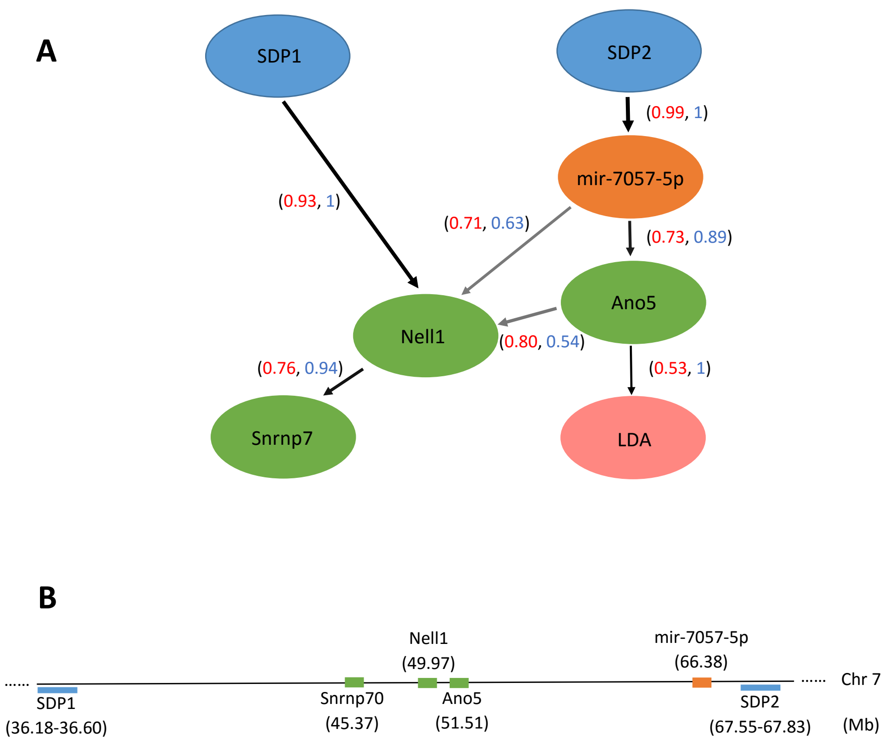

Figure 6 Bayesian network for LDA with bootstrap proportions. Figure 4 from main paper with bootstrap proportions indicated next to each edge. Red denotes the proportion corresponding to presence of the edge, blue denotes the proportion corresponding to direction of the edge.

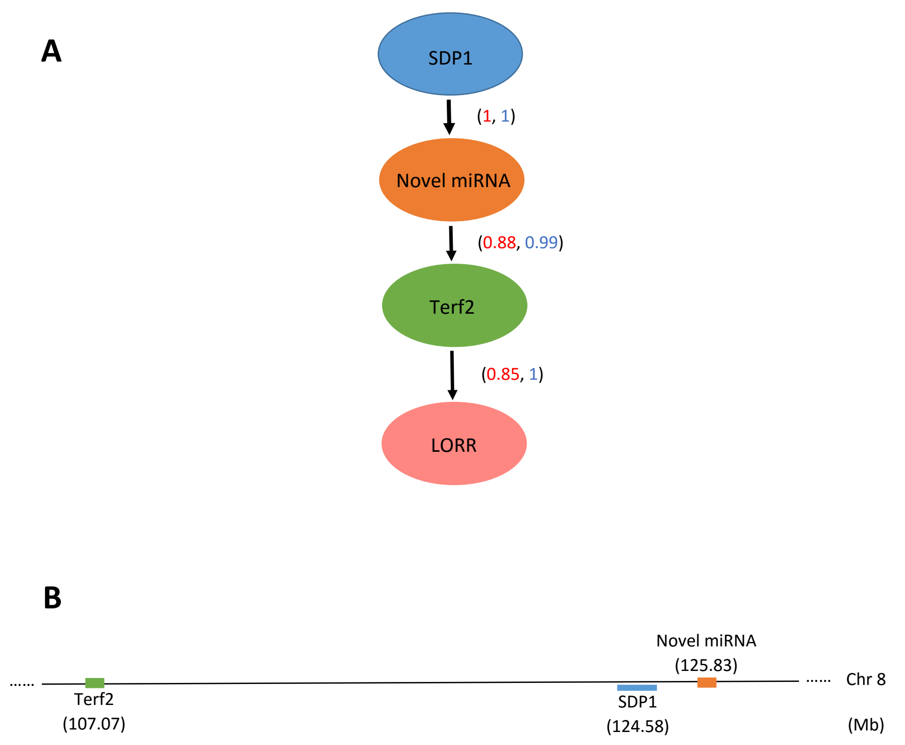

Figure 7 Bayesian network for LORR with bootstrap proportions. Figure 5 from main paper with bootstrap proportions indicated next to each edge. Red denotes the proportion corresponding to presence of the edge, blue denotes the proportion corresponding to direction of the edge.
